# Predictive factors of c-TESE success in eastern algerian patients with non-obstructive azoospermia: role of histology, hormones, and testicular volume

**DOI:** 10.1186/s12610-026-00311-7

**Published:** 2026-04-15

**Authors:** Besma Hibat allah Nourine, Leyla Ounis, Malak Hamoul, Fatima Zohra Haddad, Abdelali Zoghmar

**Affiliations:** 1 Laboratory of Molecular and Cellular Biology, Mentouri Brothers, University of Constantine I, Constantine, Algeria; 2 Department of Biochemistry and Biological Cellular and Molecular, Faculty of Nature and Life Sciences, University of Brothers Mentouri, Constantine, Algeria; 3 Ibn Rochd Clinic, Center for Reproductive Medicine, Constantine, Algeria

**Keywords:** Testicular histology, Sperm retrieval predictors, Non-obstructive azoospermia, Testicular biopsy

## Abstract

**Background:**

Non-obstructive azoospermia is the most severe and common form, representing 10–15% of infertile men. For these patients, testicular sperm extraction followed by intracytoplasmic sperm injection offer the only viable fertility option. However, sperm retrieval rates in non-obstructive azoospermia remain significantly lower than in obstructive azoospermia. Optimizing testicular sperm extraction outcomes in non-obstructive azoospermia requires understanding the clinical, hormonal, genetic, and histological factors that influence sperm retrieval. The present study aims to evaluate the impact of key clinical and biological factors on sperm retrieval out comes in patients with non-obstructive azoospermia from eastern Algeria, with the goal of improving retrieval rates and identifying reliable predictive factors for successful sperm recovery.

**Result:**

Sperm retrieval was successful in 38.3% of patients with non-obstructive azoospermia. Testicular histology had a significant impact on retrieval outcomes (*p* < 0.001). The highest sperm retrieval rates were observed in patients with late maturation arrest and hypospermatogenesis. In contrast, severe histological patterns, particularly testicular fibrosis and Sertolicell-only syndrome, were strongly associated with sperm retrieval failure, with odds ratios of 0.024 and 0.020, respectively.

Serum follicle-stimulating hormone and luteinizing hormone levels were significantly correlated with sperm retrieval success (*p* = 0.004 and *p* = 0.05, respectively). Logistic regression analysis showed an odds ratio of 0.947 for FSH, with a cut-off value of 8.83 mIU/mL, indicating that lower FSH levels were associated with higher retrieval success. For LH, the odds ratio was 1.069, with a cut-off value of 5.75 mIU/mL.

**Conclusion:**

Overall, testicular histology, serum follicle-stimulating hormone levels, and testicular volume emerged as the most important independent predictors of sperm retrieval success in Algerian patients with non-obstructive azoospermia undergoing testicular sperm extraction. Luteinizing hormone was identified as a weaker predictive factor compared with FSH and testicular volume.

Although, histological evaluation is obtained post-biopsy, it remains clinically valuable by guiding personalized patient management, including the selection of the most appropriate sperm retrieval technique, the initiation of hormonal therapy, and the prediction of outcomes in subsequent retrieval attempts.

## Résumé

**Contexte:**

L’azoospermie non obstructive (ANO) est la forme la plus sévère et la plus courante, présente chez 10 à 15 % des hommes infertiles. Pour ces patients, l’extraction testiculaire de spermatozoïdes suivie d’une injection intracytoplasmique de spermatozoïdes représente la seule option viable de fertilité. Cependant, les taux de prélèvement de spermatozoïdes dans l’ANO restent significativement plus faibles que dans l’azoospermie obstructive. Optimiser les résultats d’extraction des spermatozoïdes testiculaires lors d’une ANO nécessite de comprendre les facteurs cliniques, hormonaux, génétiques et histologiques qui influencent la récupération des spermatozoïdes. La présente étude vise à évaluer l’impact des principaux facteurs cliniques et biologiques sur les résultats de prélèvement de spermatozoïdes chez les patients atteints d’ANO provenant de l’est de l’Algérie, dans le but d’améliorer les taux de prélèvement et d’identifier des facteurs prédictifs fiables pour une récupération réussie des spermatozoïdes.

**Résultats:**

La récupération de spermatozoïdes a été réussie chez 38,3 % des patients atteints d’ANO. L’histologie testiculaire a eu un impact significatif sur les résultats de la ponction (p < 0,001). Les taux de prélèvement de spermatozoïdes les plus élevés ont été observés chez les patients présentant un arrêt tardif de maturation et une hypospermatogenèse. En revanche, les profiles histologiques sévères, en particulier la fibrose testiculaire et le syndrome des cellules de Sertoli seules, étaient fortement associés à l’échec du prélèvement de spermatozoïdes, avec des odds ratios respectivement de 0,024 et 0,020. Les taux sériques de FSH et de LH étaient significativement corrélés au succès du prélèvement des spermatozoïdes (respectivement p = 0,004 et p = 0,05). L’analyse de régression logistique a montré un odds ratio de 0,947 pour la FSH, avec une valeur seuil de 8,83 mIU/mL, indiquant que des niveaux plus faibles de FSH étaient associés à un succès de la ponction plus élevé. Pour la LH, l’odds ratio était de 1,069, avec une valeur seuil de 5,75 mIU/mL.

**Conclusion:**

Dans l’ensemble, l’histologie testiculaire, les niveaux de FSH sériques et le volume testiculaire sont apparus comme les principaux prédicteurs indépendants du succès de la récupération de spermatozoïdes chez les patients atteints d’ANO lors d’une extraction de spermatozoïdes testiculaires. La LH a été identifiée comme un facteur prédictif plus faible que la FSH et le volume testiculaire. Bien que l’évaluation histologique soit réalisée après la biopsie, elle reste cliniquement précieuse en guidant une prise en main personnalisée du patient, incluant le choix de la technique de prélèvement la plus appropriée, et la prédiction des résultats lors des tentatives ultérieures.

**Mots-clés:**

Histologie testiculaire, Prédicteurs du Prélèvement de Spermatozoïdes, Azoospermie non obstructive, Biopsie testiculaire

## Introduction

Azoospermia is defined as the Complete absence of spermatozoa in the ejaculate, confirmed on at least two separate men analyses [1]. Unlike obstructive azoospermia (OA), non-obstructive azoospermia (NOA) is the most severe and prevalent form of the condition, accounting for approximately 10–15% of cases among infertile men. For these individuals,

Assisted reproductive technologies (ART) offer the only viable solution, with surgical sperm retrieval methods such as Testicular Sperm Extraction (TESE) being the primary approach. If sperm retrieval is successful, the extracted sperm can then be used to proceed with Intracytoplasmic Sperm Injection (ICSI) [2]. In patients with OA, sperm retrieval rates (SRR) are close to 90.6% and continue to be much higher than in individuals with NOA, whose success rates are 57.6% [3]. This highlights the importance of identifying the factors that influence sperm retrieval (SR) in NOA, with the goal of optimizing TESE outcomes through personalized therapeutic strategies and medical interventions, thereby improving the overall prognosis like the choice of surgical technique, with microdissection TESE (micro-TESE) offering higher retrieval rates.

Finding and improving prognostic markers is crucial for SR results in patients with NOA, especially in advance of TESE. Numerous genetic, hormonal, and clinical factors are at play. The chances of success can be impacted by clinical factors such as the patient's age, testicular volume, medical history, and the existence of varicocele. The prognosis is greatly influenced by histology patterns such as Sertolicell-only syndrome (SCO), maturation arrest (MA), or hypospermatogenesis (HS), as well as genetic variables such Y-chromosome microdeletions or Klinefelter syndrome [4, 5]. For NOA patients, testicular histology is categorized into four main patterns, ranked from the most to the least productive. The first pattern is HS, where all stages of spermatogenesis are present, but the overall number of mature spermatozoa is significantly reduced. The second pattern, maturation arrest, is defined by an interruption of spermatogenesis at a distinct developmental stage, most frequently at the spermatogonial or primary spermatocyte stage; these are respectively named early and late maturation arrest. SCO involves the complete absence of germ cells, with seminiferous tubules comprising only Sertoli cells. Lastly, tubular hyalinization (TH) is characterized by the replacement of seminiferous tubules with fibrotic or hyalinized tissue and the absence of both germ and Sertoli cells [6]. The identification of the histological profile has been demonstrated to be an effective and proven predictive factor for SRR [7],

Alongside other factors, hormonal evaluations (including follicle-stimulating hormone (FSH), luteinizing hormone (LH), and testosterone (TST) levels), which can influence testicular histology, help predict testicular outcomes and guide the choice of sperm retrieval technique [8]. After FSH was established as a traditional predictor of TESE success, testosterone and LH levels have also been described as emerging predictive markers [9]. Among traditional predictors, testicular volume is also described as a non-invasive marker, as only testicular volume and patient age remain significantly associated with successful SR [8].

In view of providing adequate and personalized care for patients from eastern Algeria, and considering the importance of these various factors in the choice of technique and especially in predicting sperm retrieval rate in this population, this study was conducted with the objective of assessing the impact of these factors on the outcomes of sperm retrieval techniques in patients of Algerian origin.

### Patients and methods

#### Type of study

We conducted a retrospective study of medical records from patients who attended the ART center at the IBN ROCHD Clinic in Constantine between 2018 and 2025. Among these cases, 128 patients diagnosed with azoospermia were selected for further analysis.

#### Patients

**Fig. 1:**
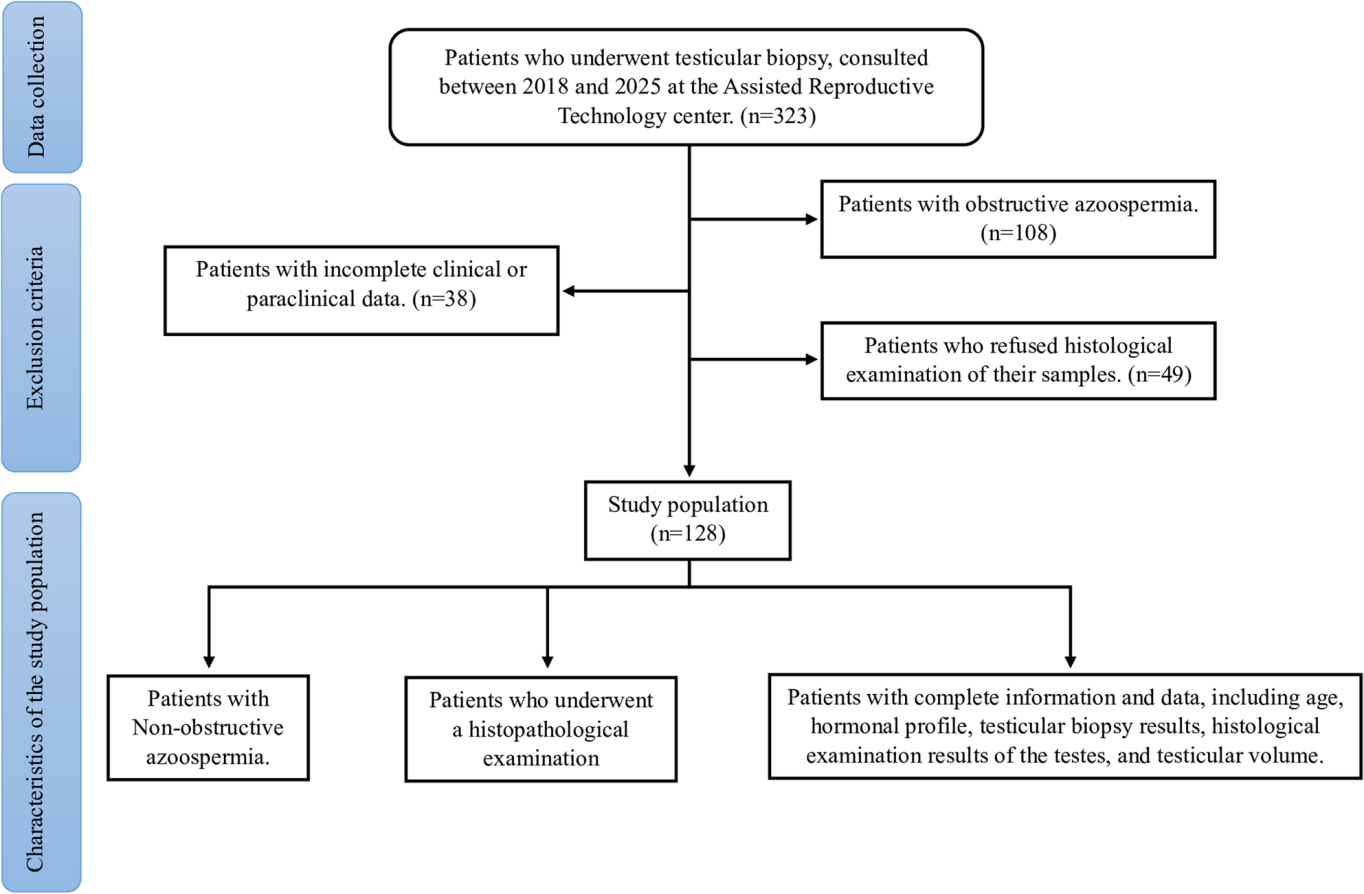
Study Flowchart Illustrating Patient Inclusion and Exclusion. Legend: A total of 323 patients who underwent testicular biopsy between 2018 and 2025 at the Assisted Reproductive Technology center were initially assessed. Patients with obstructive azoospermia, incomplete clinical or paraclinical data, or refusal of histological examination were excluded. The final study population consisted of 128 patients with non-obstructive azoospermia who had complete clinical, hormonal, and histopathological data

All patients included in the study underwent testicular biopsy to investigate the underlying causes of their infertility. Only those who had a complete clinical file, including histopathological examination, hormonal evaluation (FSH, LH, TST), and testicular ultrasound, were included.Patients were excluded from the study based on the following criteria:those diagnosed with OA; patients who did not undergo a testicular biopsy; individuals who did not have a histopathological examination;those whose medical records lacked essential clinical or personal data such as age, hormonal profile, and testicular volume as determined by ultrasonography; and patients who declined to respond to the questionnaire.Patients with OA, those who did not undergo a testicular biopsy, individuals without histopathological examination results, patients with incomplete medical records lacking essential clinical or personal data, and those who refused to complete the questionnaire were excluded from the study. The selection, inclusion, and exclusion criteria are illustrated in the flow diagram shown in Fig. 1.

#### Testicular histology

The testicular histopathological examination involves the microscopic analysis of tissue samples obtained through biopsy, assessing both histological and cytological features. This evaluation offers a thorough insight into the functional and structural condition of the testis.

#### Conventional Testicular Sperm Extraction (TESE)

The conventional testicular biopsy is a straightforward surgical procedure commonly performed in clinical settings to extract spermatozoa, particularly in men with NOA. The procedure begins with a small 1 to 2 cm incision on the scrotal skin. The paratesticular space is then opened to expose the lateral surface of the testis. An incision is made on the tunica albuginea, followed by gentle pressure applied to the testis to extrude the testicular tissue, which is carefully harvested using fine scissors. At the end of the procedure, each tissue layer is closed with absorbable sutures.

#### Statistical analysis

The data used in this study were collected from the medical records of the included patients. Rigorous data entry was performed using Microsoft Access 2016 and Microsoft Excel 2016 to create a structured and usable database.

Data processing and statistical analysis were carried out using SPSS software, version 23. Variables were analyzed according to the studied parameters. For qualitative variables, the chi-square test (χ^2^) was employed to detect statistically significant differences between various factors and the observed histological types. The differences were described as significant if *p* < 0.05.

Multivariate logistic regression analysis was performed to identify independent predictors of successful sperm retrieval. The dependent variable was sperm retrieval outcome, defined as successful or unsuccessful sperm retrieval during TESE. Independent variables included age, FSH and LH levels, and testicular histological type. Hypospermatogenesis was used as the reference category as it is considered the histological pattern closest to normal spermatogenesis due to the presence of all stages of germ cell development, albeit at reduced levels compared to normal. All variables were entered simultaneously into the model. Results were expressed as odds ratios (ORs) with 95% confidence intervals (CIs). A *p*-value < 0.05 was considered statistically significant.

## Results

The mean patient age was 39.23± 7.179 years (26–62 years), with 57.8% of patients under 40 years. SR was positive in 49 patients (38.3%), while varicocele was absent in 97 cases (75.8%). All the following parameters were evaluated according to SR and most of them showed a correlation.

### The influence of histology of testies on the SR results

Sertoli Cell-Only syndrome was diagnosed in 57 patients (44.5%). Tubular hyalinization was found in 12 patients (9.4%), while early and late maturation arrest were observed in 24 (18.8%) and 20 (15.6%) patients, respectively. Hypospermatogenesis was identified in 15 patients (11.7%).

A significant difference was found between histological types and SR (*p* = 0.000). The histological patterns most frequently associated with a positive testicular biopsy outcome are late maturation arrest and hypospermatogenesis. Conversely, negative biopsy results are mostly observed in the presence of SCO. This strong significance highlights the role of testicular histology and histological examinations in influencing SR outcomes and in predicting the results (Table 1).

**Table 1:** Association between histological patterns and successful SR in NOA patients

Histological pattern	Successful SR *N* (%)	No sperm retrieved *N* (%)	*p*-value
SCO	12 (24.5)	45 (57)	0.000***
HYPO	14 (28.6)	1 (1.3)	
TH	3 (6.1)	9 (11.4)	
MAE	6 (12.2)	18 (22.8)	
MAL	14 (28.6)	6 (7.6)	
Total	49 (100)	79 (100)	

Presents the relationship between testicular histological patterns and sperm retrieval outcomes in the studied cohort. Data are expressed as number of patients (N) and percentage (%). Patients with HYPO and MAL patterns showed higher sperm retrieval rates, while those with SCO and MAE had lower rates. Statistical significance was assessed using the Chi-square test. A *p*-value < 0.05 was considered significant

*Abbreviations*: *SR* Sperm Retrieval, *SCO* Sertoli Cell-Only syndrome, *HYPO* Hypospermatogenesis, *TH* Tubular hyalinization, *MAE* Early Maturation Arrest, *MAL* Late Maturation Arrest

***p < 0.001

### The influence of hormones levels on the SR results

The mean serum levels of FSH, LH, and testosterone were 15.65 mIU/mL, 9.49 mIU/mL and 5.75 ng/mL respectively, with the majority of values falling within the normal range for each hormone.

#### FSH

In our study population, 53.1% of patients had FSH levels within the normal range [1.7–12 mIU/mL], 1.6% had decreased levels [< 1.7 mIU/mL], and 45.3% had elevated levels [> 12 mIU/mL]. FSH values ranged from a minimum of 1.00 to a maximum of 78.30.

The group of patients with normal FSH levels achieved a higher rate of positive testicular biopsy results, While the highest rate of negative SR was observed in the group with elevated FSH levels. A significant difference was found between FSH levels and TESE outcomes (*p* = 0.004) (Table 2).

**Table 2:** Comparison of hormone levels according to SR outcomes

Hormone levels		Successful SR *N* (%)	No sperm retrieved *N* (%)	*p*-value
FSH (mIU/mL)	< 1.7	2 (4.1)	0 (0)	0.004*
	1.7–12	33 (67.3)	35 (44.3)	
	> 12	14 (28.6)	44 (55.7)	
Total		49 (100)	79 (100)	
LH (mIU/mL)	< 5	22 (44.9)	21 (26.6)	0.050
	5–10	17 (34.7)	28 (35.4)	
	> 10	10 (20.4)	30 (38.0)	
Total		49 (100)	79 (100)	
TST ng/mL	< 3	10 (20.4)	27 (34.2)	0.248
	3–10	33 (67.3)	44 (55.7)	
	> 10	6 (12.2)	8 (10.1)	
Total		49 (100)	79 (100)	

Shows the distribution of hormone levels according to the outcome of sperm retrieval in the studied cohort. Data are presented as number of patients (N) and percentage (%). Hormone levels include FSH, LH, TST. Statistical comparisons were performed using the Chi-square test. A *p*-value < 0.05 was considered statistically significant

*Abbreviations*: *FSH* Follicle-stimulating hormone, *LH* Luteinizing hormone, and TST: total testosterone, *SR* Sperm Retrieval

^*^p < 0.05

#### LH

For LH, 35.2% of the population had normal levels [5- 10 mIU/mL], while low [< 5 mIU/mL] and elevated [> 10 mIU/mL] LH were recorded in 33.6% and 31.3%, respectively. The minimum recorded value was 0.12 and the maximum was 45.82 mIU/mL.

A positive TESE result was observed in 44.9% of patients with low LH levels, whereas the group with elevated LH levels exhibited the highest rate of negative SR. No statistically significant association was found between LH levels and TESE outcomes (*p* = 0.05) (Table 2).

#### TST

Patients with normal testosterone levels [3–10 ng/mL] represented 60.2% of our population, followed by 28.9% with low testosterone levels [< 3 ng/mL] and 10.9% with elevated levels [> 10 ng/mL]. The lowest recorded value was 0.17 and the highest was 66.17 ng/mL.

A majority of patients with normal testosterone levels had positive TESE results, The same group also showed the highest rate of negative SR; however, no statistically significant association was found between testosterone levels and TESE outcomes(*P >* 0.05) (Table 2).

### The influence of testicular volumes on the SR results

Normal testicular volume [10–15 ml] was observed in 63.6% of the cases, followed by hypotrophic volumes [5–10 ml] in 31.3%, and atrophic volumes [< 5 ml] in 3.3% of the cases.

Patients with normal testicular volume represented the group with the highest rate of positive SR following biopsy. In contrast, patients with hypotrophic and atrophic testicular volumes showed a lower success rate. However, due to the large number of patients with normal testicular volume, this group also accounted for the highest proportion of negative SR outcomes, followed closely by the hypotrophic group, which accounted for 40.5% (Table 3).

**Table 3:** Comparison of Testicular volumes categories according to SR outcomes

		Successful SR *N* (%)	No sperm retrieved *N* (%)	*P* value
Testicular volumes	Atrophic [< 5 ml]	3 (6.1)	4 (5.1)	0,016*
	Hypotrophic [5–10 ml]	8 (16.3)	32 (40.5)	
	Normal [10–15 ml]	38 (77.6)	43 (54.4)	
Total		49 (100)	79 (100)	

Shows the distribution of testicular volume categories according to the outcome of sperm retrieval in the studied cohort. Data are presented as number of patients (N) and percentage (%). Testicular volumes were classified as atrophic (< 5 mL), hypotrophic (5–10 mL), and normal (10–15 mL). Statistical comparisons were performed using the Chi-square test. A *p*-value < 0.05 was considered statistically significant

*Abbreviations*: *SR* Sperm Retrieval

^*^p < 0.05

### Predictors of SR success

In the multivariate logistic regression analysis, age was not significantly associated with SR success. The FSH level was significantly associated with SR outcome, with higher FSH values reducing the likelihood of successful SR, and higher LH levels were positively associated with SR success.

Regarding histological type, hypospermatogenesis was used as the reference category.

Early maturation arrest was significantly associated with lower odds of successful SR. Similarly, testicular fibrosis and Sertoli Cell-Only Syndrome were independently associated with markedly reduced odds of SR. In contrast, no statistically significant association was observed for late maturation arrest (Table 4). In summary, multivariate analysis identified FSH level, LH level, early maturation arrest, testicular fibrosis, and Sertoli Cell-Only Syndrome as independent predictors of sperm retrieval success, while age and late maturation arrest did not retain statistical significance after adjustment.

**Table 4:** Multivariate logistic regression analysis of factors associated with successful SR

Variables	OR	CI 95%	P (Sig.)
Age (years)	1.058	[0.995–1.126]	0.073
FSH	0.947	[0.900–0.996]	0.034*
LH	1.069	[1.001–1.142]	0.048*
Histological type (Late MA vs Hypospermatogenesis)	0.157	[0.016–1.513]	0.109
Histological type (Early MA vs Hypospermatogenesis)	0.023	[0.002–0.224]	0.001**
Histological type (Fibrosis vs Hypospermatogenesis)	0.024	[0.002–0.348]	0.006*
Histological type (SCO vs Hypospermatogenesis)	0.020	[0.002–0.177]	< 0.001***

Shows the results of multivariate logistic regression analysis assessing the factors associated with successful sperm retrieval. Odds ratios (OR) and 95% confidence intervals (CI) are presented for each variable. Age, FSH, LH and histological type were included in the model. SR: Sperm Retrieval; Statistical significance was considered at *p* < 0.05

*Abbreviations*: *FSH* Follicle-Stimulating Hormone, *LH* Luteinizing Hormone, *SR* Sperm Retrieval, *SCO* Sertoli Cell-Only syndrome, *MA* Maturation Arrest

^*^p < 0.05

^**^p < 0.01

^***^p < 0.001

We developed a predictive model for SR based on logistic regression analysis, aiming to identify factors associated with successful SR in NOA patients undergoing TESE.

**Fig. 2:**
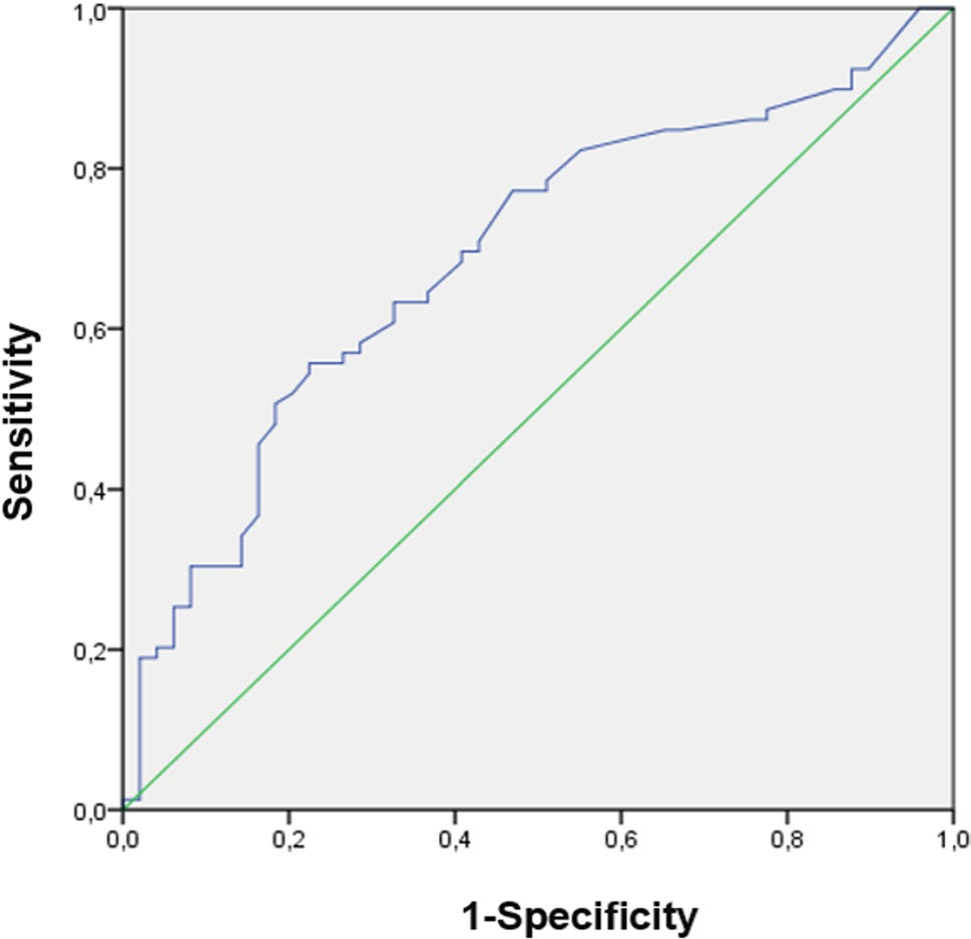
Receiver operating characteristic (ROC) curve illustrating the diagnostic performance of FSH levels for predicting successful sperm retrieval. Legend: This ROC curve shows the relationship between sensitivity and 1-specificity for different FSH cut-off values. The optimal FSH cut-off value was 8.83 mIU/mL, yielding a sensitivity of 69.6% and a specificity of 59.2% for predicting successful sperm retrieval. FSH: Follicle-Stimulating Hormone

**Fig. 3:**
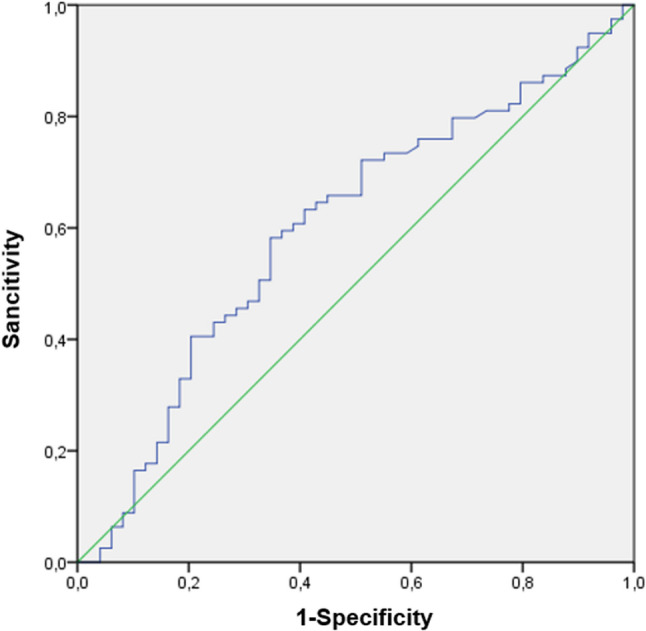
Receiver operating characteristic (ROC) curve illustrating the diagnostic performance of LH levels for predicting successful sperm retrieval. Legend: This ROC curve shows the relationship between sensitivity and 1-specificity for different LH cut-off values. The optimal LH cut-off value was 5.82 mIU/mL, yielding a sensitivity of 64.6% and a specificity of 57.1% for predicting successful sperm retrieval. LH: Luteinizing Hormone. ROC: Receiver operating characteristic

- Therefore, we identified an FSH cut-off value of 8.83 mIU/mL, demonstrating a sensitivity of 69.6% and a specificity of 59.2% (Fig. 2). Patients with FSH levels above the reference value showed a lower rate of successful sperm retrieval and a higher proportion of failure. In contrast, patients with FSH levels within or below the reference range demonstrated a higher likelihood of successful sperm retrieval compared to negative outcomes (Table 5).
- The cut-off value is 5.82 mUI/ml with a calculated specificity of 57.1% and sensitivity of 64.6%for the LH. Suggesting a potential predictor of successful SR in TESE procedures (Fig. 3). Among patients with LH levels above the reference threshold, achieved successful SR, whereas 70.8% experienced retrieval failure. Conversely, patients with LH levels below the reference cut-off demonstrated a higher likelihood of successful SR (Table 6).

**Table 5:** Comparison of FSH cut-off values according to SR outcomes

		Successful SR*N* (%)	No sperm retrieved *N* (%)
FSH cut off (mIU/mL)	> 8.83	20 (26.7)	55 (73.3)
	< 8.83	29 (54.7)	24 (45.3)

Shows the number and percentage of patients with successful or unsuccessful sperm retrieval according to FSH cut-off values. The cut-off value was set at 8.83 mIU/mL. This table allows comparison of sperm retrieval outcomes between patients with FSH levels above and below the cut-off

*Abbreviations*: *SR* Sperm Retrieval, *FSH* Follicle-Stimulating Hormone

**Table 6:** Comparison of LH cut-off values according to SR outcomes

		Successful SR *N* (%)	No sperm retrieved *N* (%)
LH cut off (mIU/mL)	> 5.75	21 (29.2)	50 (70.8)
	< 5.75	28 (50)	28 (50)

Shows the number and percentage of patients with successful or unsuccessful sperm retrieval according to LH cut-off values. The cut-off value was set at 5.75 mIU/mL. This table allows comparison of sperm retrieval outcomes between patients with LH levels above and below the cut-off

*Abbreviations*: *SR* Sperm Retrieval, *LH* Luteinizing Hormone

### The influence of hormones levels on the testicular histology

There was a statistically significant difference between the various histological types and the hormone levels analyzed. FSH and LH levels showed a significant association (*p* = 0.000), while testosterone levels were also significantly associated (*p* = 0.012). A significant difference was observed between testicular volumes and the different histological types (*p* = 0.003).

## Discussion

In this study, three factors were identified as predictive of successful sperm retrieval (SRR) in Algerian patients, aiming to establish a prognostic system to guide assisted reproductive procedures through personalized care. Our results demonstrate that testicular histological type is a major determining factor in the success of SR in patients with NOA.

Among the different histological profiles observed, late maturation arrest proved to be the most favorable, with a positive SRR. This histological pattern is characterized by partially preserved spermatogenesis, with germ cell development progressing to advanced stages (secondary spermatocytes or early spermatids) before being interrupted. Such residual spermatogenic activity may explain the presence of viable spermatozoa in certaines areas of the testicular tissue. These findings are consistent with previous reports describing SRR ranging from 20 to 40% in patients with late maturation arrest, likely due to the presence of focal areas of preserved spermatogenesis [10].

Hypospermatogenesis demonstrated comparable SR outcomes due to the presence of all germ cell lineages, albeit in reduced numbers, reflecting a quantitative decrease in sperm production rather than a complete absence. This indicates that spermatogenesis still occurs, but fewer sperm are produced overall. Certain studies have shown that hypospermatogenesis generally yields the highest SRR, often exceeding 40% [11].

In contrast, testicular fibrosis showed a very low SR success rate, and the multivariate logistic regression analysis supports this finding, demonstrating that testicular fibrosis was independently associated with poor SR outcomes compaired to hypospermatogenesis. Which can be attributed to severely impaired spermatogenesis resulting from major disruption of seminiferous tubule architecture and extensive loss of germ cells, often accompanied by replacement of interstitial tissue with fibrous connective tissue. Testicular fibrosis has been reported to be associated with low SR rates, often below 10%, due to irreversible degeneration of the germinal epithelium [12]. Early maturation arrest was associated with successful SR in only 12.2% of patients and showed a markedly reduced likelihood of SR compared with hypospermatogenesis. This reflects early disruption of germ cell development and limited residual spermatogenic activity [13].

The most unfavorable histological type for SR remains Sertoli Cell-Only Syndrome. Due to its embryological origin, SCO is characterized by an almost complete absence of germ cells, with seminiferous tubules containing only Sertoli cells. This pattern reflects irreversible damage to the germ cell lineage, making sperm production virtually impossible. Reported as the most severe form, SCO exhibits markedly lower SRR compared to other histological types, generally around 20–30%, and sometimes below 10% in more severe series [14].

Some studies have also found a strong correlation between testicular histology and SRR in men with NOA, while SCO is linked to the lowest success rates [15, 16]. Testicular histology is thus considered a good predictor of TESE outcomes [17]. However, although correlations have been demonstrated, some caution remains necessary, as in certain cases (30%) the prediction of the outcome based on histology remains limited, leading to the conclusion that the correlation is only partial [18, 19].

From a hormonal perspective, FSH was found to be a marker significantly associated with TESE outcomes and the observed histological types. This can be attributed to FSH signaling is essential for the initiation and progression of spermatogonial proliferation and differentiation, regulating germ cell survival and entry into meiosis via Sertoli cell-mediated mechanisms.

In our study, 55.7% of the patients had elevated FSH levels and a negative TESE outcome. This increase in FSH can be explained by a decrease in inhibin B secretion, secondary to severe testicular damage. This disruption leads to a loss of negative feedback at the pituitary level, resulting in FSH overproduction. Conversely, patients with normal FSH levels showed favorable SR outcomes. However, only two patients presented with low FSH levels, making the results for this category inconclusive so high FSH levels are negatively associated with the success of TESE. The multivariate logistic regression analysis confirmed this finding, with higher FSH values reducing the likelihood of successful SR.

In this context, FSH has previously been associated with TESE outcomes, as FSH levels were significantly higher in patients with no SR, showing a statistically significant difference (*P* < 0.001). Moreover, FSH was negatively correlated with TESE success (*P* < 0.001) [20]. Thus, higher FSH levels were associated with a reduced likelihood of SR [21]. An increase of one unit in FSH was associated with a 10% higher risk of SR failure [22]. FSH is often considered a predictive marker for SR outcomes. For this reason, FSH levels are used as an indicator to guide the choice of SR technique, with patients presenting high FSH levels being directed toward micro-TESE in order to optimize the SRR [23].

Receiver Operating Characteristic (ROC) curve analysis identified a cut-off value of 8.83 mIU/mL. Below this threshold, the positive biopsy rate reached 57.7%. Thus, FSH can be considered a relevant predictive factor for the success of TESE. Other studies have identified a cut-off value around 14 mIU/mL, this difference suggests that the optimal FSH threshold may vary across populations and clinical settings. Although this cut-off is higher than the one observed in our population, their ROC curves demonstrated higher sensitivity and specificity [22]. The difference between the FSH cutoff observed in our study and the cutoff commonly reported in the literature (~ 14 mIU/mL) can be explained by several factors. First, our study included a broader range of histological patterns, whereas other studies often focus on fewer or more severe patterns. Second, the mean FSH levels in our population were lower than in other cohorts, which generally include patients with more severe phenotypes, resulting in higher average FSH levels and, consequently, a higher cutoff. Third, the SR technique used also influences outcomes: our study employed conventional TESE, while other studies often include micro-TESE, which is known to increase the likelihood of SR. These differences likely explain the lower FSH cutoff observed in our study compared with previous reports. Despite the different genetic backgrounds of the patients with NOA included in our study compared with those in previous studies, these discrepancies are primarily due to the lack of universal data in this field regarding the Algerian population. Taken together, these considerations support the credibility of our FSH threshold, taking into account the differences in clinical criteria, patient population, and SR technique used in our study [24–26].

Statistical analysis demonstrated a trend toward an association between LH levels and TESE outcomes (*p* = 0.050), indicating a weak correlation. However, multivariate analysis showed a positive association between LH levels and SR outcomes (*p* < 0.05) confirming its clinical relevance when accounting for other factors. The LH levels showed a correlation with histological testicular profiles (*P* < 0.001), this may be attributed to the fact that LH stimulates Leydig cells to produce testosterone, which is essential for spermatogenesis; however, LH does not directly regulate the proliferation and maturation of germ cells.

Elevated LH levels were associated with the lowest SR success rate. For the low and normal LH level categories, the results are very similar, favoring a positive SR. The multivariate logistic regression analysis showed that higher LH levels were associated with a modest but statistically significant increase in the likelihood of SR.

Although this factor is frequently described as having limited predictive accuracy for TESE outcomes, several studies have nonetheless demonstrated a statistically significant correlation [27]. Notably, lower LH levels have been associated with improved success rates [28, 29].

Analysis using the ROC curve identified a discriminative cutoff value of 5.83 mIU/mL. Above this threshold, negative biopsies accounted for 70.8%. Conversely, below this cutoff, the proportion of positive biopsy results increased significantly. According to the literature, different thresholds have been identified, with highly variable rates ranging between 2 mIU/mL and 13.1 mIU/mL for NOA patients [28, 30], and reaching up to 21 mIU/mL for other conditions such as Klinefelter syndrome [31]. LH primarily reflects Leydig cell function, whereas spermatogenesis mainly depends on Sertoli cells, which are better assessed by other parameters such as FSH levels and histological analysis. In severe histological patterns, such as testicular fibrosis or SCO, LH levels may remain within the normal range despite the absence of spermatogenesis, which weakens the association between LH and SR outcomes. Consequently, LH remains a weak predictive factor and has often been reported as a dependent rather than an independent predictor, for example in relation to other hormones such as testosterone [28]. The T/LH ratio, FSH levels, and bilateral testicular volume were independent predictors of successful SR. The balance between testosterone and LH, which reflects Leydig cell efficiency and the overall hormonal support for spermatogenesis, may be a more sensitive indicator of testicular function than LH alone. The T/LH ratio cutoff determined by ROC analysis was 0.68, with a sensitivity of 72.1% and a specificity of 81.4% [10].

Regarding testosterone, no statistically significant difference was observed between its levels and TESE outcomes, since testosterone is primarily involved in the later stages of spermatogenesis, it does not directly influence the presence or absence of spermatozoa in the testis. In our cohort, the majority of patients had testosterone levels within the normal range.

It has been reported that testosterone levels do not allow for the prediction of the presence or absence of testicular spermatozoa, as Leydig cell function may be preserved while Sertoli cell function is impaired. Consequently, circulating testosterone levels can remain normal even in cases of spermatogenic failure [32]. Although generally considered a weak predictive marker, testosterone has been statistically correlated with TESE outcomes in some studies (*p* = 0.021). This association may be explained by the hormone's contribution to creating a more favorable intratesticular microenvironment for spermatogenesis [33]. The presence of statistical significance in FSH and LH highlights the important role of these hormones in the early stages of spermatogenesis, while the absence of significance for testosterone remains plausible given its effect mainly occurs during the later stages of spermatogenesis.

Testicular volume showed a significant association with TESE outcomes as it reflects the functional mass and the spermatogenic environment. Normal testicular volume appears to be the most favorable factor for SR. It shows the highest success rate compared to the other categories. Although patients with normal volume a high rate of the negative outcomes due to their larger numbers, they are closely followed by the hypotrophic group. This represents a notably high failure rate compared to their relatively low overall success rate.

Testicular volume has long been recognized in the literature as a predictive factor for SR success in TESE procedures [34]. A smaller testicular volume is associated with SR failure, as a larger volume generally reflects a greater amount of active testicular tissue and thus a higher likelihood of finding viable spermatozoa during biopsy or TESE. In contrast, reduced testicular volume often indicates germinal tissue loss or atrophy. Therefore, testicular volume is considered to be correlated with both the quality and quantity of germ cells [28, 35]. However, more recently, testicular volume has been suggested not to be considered an independent predictive factor, as individual variability and histological differences must be taken into account when evaluating these parameters [20].

### Limitations of the study

This study is a single-center study with a relatively small sample size compared with other studies, which may limit the generalizability of the findings. A larger sample would have been more informative and comparable to studies conducted by our peers. Micro-TESE was not included due to limited data, as this technique was only recently introduced into our protocols. Additionally, a genetic component could have been included, but this was not feasible due to the unavailability of such analyses in our country. Furthermore, as previously mentioned, karyotyping is not a routine analysis in our clinical practice for all patients, meaning that some cases of Klinefelter syndrome may not have been systematically detected. Moreover, hormonal parameters such as inhibin B and AMH could not be included in our study due to limited data, as the availability of these analyses is very restricted and their cost is high. We also wish to note that, during the evaluation of testicular volume by ultrasound, the presence or absence of small hyperechoic foci was neither recorded nor reported in this study. Nevertheless, this study represents the first comprehensive work on the eastern Algerian population, addressing the lack of data in this region.

## Conclusion

Our study demonstrates that elevated FSH levels, hypotrophic or atrophic testicular volume, and histological profiles such as Sertoli Cell-Only Syndrome or early maturation arrest are unfavorable indicators for a successful TESE outcome. Therefore, testicular histology, testicular volume, and FSH levels are important independent predictors of TESE results and SRR in Algerian NOA patients, whereas LH remains a less influential, dependent predictive factor.

Although histological assessment is often performed after a TESE, it may still help predict the outcome of a subsequent TESE and its effectiveness. This may allow clinicians to take the necessary steps in establishing personalized care protocols, whether by choosing the most appropriate sperm retrieval technique.

## Data Availability

The data cannot be made publicly available due to [confidentiality, ethics, patient rights, institutional ownership, etc. **]** . They are available from the corresponding author upon reasonable request.

## Abbreviations

ART: Assisted reproductive technologies
χ^2^: Chi-square test
CIs: Confidence intervals
FSH: Follicle-stimulating hormone
HS: Hypospermatogenesis
ICSI: Intracytoplasmic Sperm Injection
LH: Luteinizing hormone
MA: Maturation arrest
Micro-TESE: Microdissection TESE
NOA: Non-obstructive azoospermia
ORs: Odds ratios
OA: Obstructive azoospermia
ROC: Receiver Operating Characteristic
SCO: Sertoli Cell-Only Syndrome
SRR: Sperm retrieval rates
SR: Sperm retrieval
TESE: Testicular Sperm Extraction
TH: Tubular hyalinization
TST: Testosterone
